# Activation of mechanoreceptor Piezo1 inhibits enteric neuronal growth and migration *in vitro*

**DOI:** 10.3389/fnmol.2024.1474025

**Published:** 2024-12-20

**Authors:** Chioma Moneme, Oluyinka O. Olutoye, Michał F. Sobstel, Yuwen Zhang, Xinyu Zhou, Jacob L. Kaminer, Britney A. Hsu, Chengli Shen, Arabinda Mandal, Hui Li, Ling Yu, Swathi Balaji, Sundeep G. Keswani, Lily S. Cheng

**Affiliations:** ^1^Department of Surgery, University of Virginia, Charlottesville, VA, United States; ^2^Department of Surgery, Baylor College of Medicine, Houston, TX, United States; ^3^Department of Pediatric Surgery, Texas Children's Hospital, Houston, TX, United States

**Keywords:** enteric nervous system (ENS), enteric neural crest-derived progenitor cells (ENPC), biomechanical force, mechanotransduction, neuronal cell migration, neuronal regeneration, Piezo1

## Abstract

**Introduction:**

Dysfunction of the enteric nervous system (ENS) is linked to a myriad of gastrointestinal (GI) disorders. Piezo1 is a mechanosensitive ion channel found throughout the GI tract, but its role in the ENS is largely unknown. We hypothesize that Piezo1 plays an important role in the growth and development of the ENS.

**Methods:**

Enteric neural crest-derived progenitor cells (ENPC) were isolated from adult mouse intestine and propagated in culture as neurospheres. ENPC-derived neurons were then subject to *in vitro* stretch in the presence or absence of Piezo1 antagonist (GsMTx4). Transcriptomes of stretched and unstretched ENPC-derived cells were compared using bulk RNA sequencing. Enteric neurons were also cultured under static conditions in the presence of Piezo1 agonist (Yoda1) or antagonist. Neuronal phenotype, migration, and recovery from injury were compared between groups.

**Results:**

Though stretch did not cause upregulation of Piezo1 expression in enteric neurons, both stretch and Piezo1 activation produced similar alterations in neuronal morphology. Compared to control, neurite length was significantly shorter when stretched and in the presence of Piezo1 activation. Piezo1 inhibition prevented a significant reduction in neurite length in stretched neurons. Piezo1 inhibition also led to significantly increased neuronal migration, whereas Piezo1 activation resulted in significantly decreased neuronal migration and slower neuronal recovery from injury.

**Conclusion:**

Mechanotransduction plays an important role in regulating normal GI function. Our results suggest that the Piezo1 mechanoreceptor may play an important role in the ENS as its activation leads to decreased neuronal growth and migration. Piezo1 could be an important target for diseases of ENS dysfunction and development.

## Introduction

The enteric nervous system (ENS) is a complex network of over 500 million neurons, often referred to as the “second brain,” that orchestrates innumerable significant gastrointestinal (GI) functions, including motility, secretion, absorption, immune and endocrine function (Furness, 2012; Lomax et al., 2005; Grundy and Schemann, 2005). Dysfunction of the ENS has been linked to several functional GI disorders, most famously, Hirschsprung disease. Hirschsprung disease is characterized by the failure of enteric neural precursor cells to complete their normal migration through the GI tract, resulting in varying lengths of distal aganglionic intestine. This leads to a failure of relaxation and functional obstruction of the affected bowel segment. Importantly, the proximal, normally ganglionated intestine exhibits continued dysfunction even after surgical intervention (Butler Tjaden and Trainor, 2013; Langer, 2012). The cause of this persistent dysfunction is not well-understood, but is thought to be due to latent abnormalities in the ENS (Zaitoun et al., 2013; Cheng et al., 2016a).

Piezo1, also known as *Fam38A*, is a large trimeric transmembrane ion channel. The Piezo ion channel family is primarily recognized for its involvement in mechanotransduction, which is the ability of a cell to convert mechanical stimuli into a biological signal and response. Mechanosensitive processes have been implicated in physiologic processes such as touch, proprioception, vestibular function, tissue injury, and vascular tone (Coste et al., 2010; Hamill and Martinac, 2001). Piezo mechanoreceptors are evolutionarily conserved and can be found in many animals, plants, and eukaryotic species (Coste et al., 2012). Humans, and other vertebrate species, contain two Piezo channels, Piezo1 and Piezo2. Piezo2 has been shown to be important in the mechanotransduction patterns involved in urination (Marshall et al., 2020) and gastrointestinal motility (Servin-Vences et al., 2023). Piezo1 is expressed in a myriad of different tissues including the colon, bladder, kidneys, and skin (Coste et al., 2010). While its function in the gut is not known, Piezo1 has been linked to neural crest cell migration during development (Canales Coutino and Mayor, 2021), and thus may play a role in ENS growth and development.

Understanding the complex relationship between Piezo mechanoreceptors, biomechanical force, and the ENS holds tremendous therapeutic potential for a wide range of neurogastroenteropathies, including Hirschsprung disease. We aim to describe how Piezo1 gain- and loss-of-function affect ENS development and postnatal enteric neuronal behavior.

## Methods

### Isolation of the enteric neuronal progenitor cells

Male and female C57BL/6J mice aged 3–8 weeks (Jackson Labs, Bar Harbor, ME) were used in accordance with relevant ethical guidelines and regulations from our Institutional Animal Care and Use Committee protocols. The mice were housed in a controlled environment with a 12-h light/dark cycle and provided *ad libitum* access to food and water. The longitudinal muscle with myenteric plexus (LMMP) was dissected from the small and large bowel, and enteric neural crest-derived progenitor cells (ENPC) were isolated in accordance with published protocols and propagated in culture as neurospheres (Hotta et al., 2016a,b; Cheng et al., 2016b). Experiments were performed with secondary and tertiary neurospheres.

### Localizing Piezo1 expression in the gut

Colonic tissue from the mice, as described above, was isolated, fixed in 10% formalin, and embedded in paraffin in a standard histological fashion. The tissue block was sectioned to a desired thickness of 5 μm and placed onto glass slides. After deparaffinization, the tissue sections were permeabilized with 0.3% Triton X-100. Sections were then incubated with blocking buffer to prevent non-specific binding, followed by incubation with the primary antibodies for mouse anti-Tuj1 (1:500; BioLegend, San Diego, CA), rabbit anti-Piezo1 (1:500; Proteintech, Rosemont, IL), and rabbit anti-Piezo2 (1:500; Proteintech), and then secondary antibodies appropriate for each primary antibody including goat anti-rabbit IgG peroxidase polymer detection kit (Vector Laboratories, Newark, CA) for immunohistochemistry (IHC), donkey anti-mouse Alexa-Fluor 488 (Thermofisher Scientific, Waltham, MA), goat anti-mouse Alexa-Fluor 594 (Thermofisher Scientific), and donkey anti-rabbit Alexa-Fluor 594 (Thermofisher Scientific) for immunofluorescence (IFC) staining. Slides were mounted using a mounting media containing DAPI (VectaShield Anti-fade mounting media; Vector Laboratories). Images were captured using Leica DMi8 microscope (Leica, Deerfield, IL) or Keyence BZ-X800 microscope (Keyence, Itasca, IL).

### Enteric neuronal growth in the presence of *in vitro* stretch

ENPC were plated on fibronectin-coated BioFlex plates (FlexCell, Burlington, NC) in differentiation media at a concentration of 10,000–20,000 cells/mL at 3 mL/well and allowed to differentiate in culture into enteric neurons for 7 days. After 7 days in culture, a group of ENPC-derived cells were subjected to a stretch regimen using the FX5000C Tension System or the Flex Jr Tension System (FlexCell, Burlington, NC). This computer-regulated bioreactor system uses vacuum pressure and positive air pressure to apply cyclic or static strain to cells in culture. The cells were subjected to stretch for a total of 6 h (*n* = 3). A stepwise approach was used to reach our goal of 5% sine at 0.05 Hz. The regimen was as follows: 10 min at 0–1% sine, 10 min at 1–2% sine, 10 min at 2–3%, 10 min at 3–4% sine, then 4–5% sine for the remainder of the 6 h with maximum stretch of 5%. A control group of ENPC-derived cells were cultured on BioFlex plates for 7 days but was not subject to a stretch regimen (*n* = 3). For the proliferation assay, EdU was added to the media according to manufacturer instructions before initiation of the stretch regimen. This process was repeated with the addition of DMSO vehicle (*n* = 3) or with 3 μM GsMTx4 (*n* = 3) to media 12 h prior to the stretch regimen. ENPC were plated on fibronectin-coated BioFlex plates and allowed to differentiate in culture into enteric neurons for 7 days prior to pre-treatment with GsMTx4 or vehicle and a stretch regimen. The regimen for the pre-treated cells was as follows: 10 min at 0–0.5% sine, 10 min at 0.5–1% sine, 10 min at 1–1.5%, 10 min at 1.5–2% sine, then 2–2.5% sine for the remainder of the 6 h with maximum stretch of 2.5%. Cells were then fixed and studied with immunocytochemistry for Tuj1 with a nuclear counterstain for DAPI. Proliferation was quantified using an EdU assay (Click-iT EdU cell proliferation kit; ThermoFisher Scientific). Images were captured using a Leica DMi8 microscope or a Keyence BZ-X800 microscope. Neurite length was quantified using Image J (version 1.53k; National Institutes of Health, Bethesda, MD). Between 100 and 250 cells were analyzed per experimental repetition for each condition.

### Bulk RNA-seq and analysis of differentially expressed genes in stretched ENPC

ENPC were plated on fibronectin-coated BioFlex plates (FlexCell) in differentiation media at a concentration of 10,000–20,000 cells/mL at 3 mL/well and allowed to differentiate in culture for 7 days. After 7 days in culture, a group of cells were subjected to stretch for a total of 6 h (*n* = 3) using a stepwise approach with a final stretch of 5% sine, as described above. RNA was isolated from stretched and unstretched ENPC-derived cells and bulk RNA sequencing was performed. The resultant RNA-Seq data was mapped using STAR (Dobin et al., 2013) to the mouse genome build UCSC mm10. Gene expression quantification was achieved using FEATURECOUNTS (Liao et al., 2013) against the GENCODE gene model. Differential gene expression was evaluated using the R package EdgeR (Robinson et al., 2010) and was further normalized using the R package RUVr4 (LRT RUVr). Significance was defined as a fold change exceeding 1.5× and FDR < 0.05. Volcano plots were generated using EnhancedVolcano package in the R statistical system. Enriched pathways were assessed via the over-representation (ORA) (Subramanian et al., 2005) method using the hypergeometric distribution and the MSigDB (Liberzon et al., 2015) v7.5.1 genesets. Additional gene ontology (GO) term analyses were performed using ShinyGO (Ge et al., 2020).

### Enteric neuronal growth in the presence of Piezo1 agonism and antagonism

ENPC were plated on fibronectin-coated 8-well chamber slides in differentiation media at a concentration of 10,000–20,000 cells/mL at 500 μL/well and allowed to differentiate in culture for 7 days. Cells were subject to: DMSO vehicle only control (*n* = 13); 10 μM (*n* = 3) or 20 μM (*n* = 14) of Piezo1 agonist Yoda1; 1.5 μM (*n* = 3) or 3 μM (*n* = 14) of Piezo1 antagonist GsMTx4; or 20 μM of Yoda1 in combination with 3 μM of GsMTx4 (*n* = 3). GsMTx4, Grammostola patulate mechanotoxin 4, is a tarantula spider toxin widely used to reversibly inhibit and study mechanoreceptive channels (Bae et al., 2011). Yoda1 is a chemical compound known to specifically activate Piezo1 (Syeda et al., 2015; Liu et al., 2022). Chamber slides were studied using immunocytochemistry as described above. Neurite length, neuronal morphology, and neuronal density were quantified using an automated cell segmentation package in Image J. Between 250 and 1,000 cells were analyzed per condition for each experimental repetition.

### Enteric neuronal migration in the presence of Piezo1 agonism and antagonism

To assess neuronal cell migration, undissociated neurospheres were plated on a fibronectin-coated chamber slide in differentiation media with no additional additive (*n* = 4), 20 μM Yoda1 (*n* = 6), or 3 μM GsMTx4 (*n* = 6). After 7 days, the cells were fixed and studied using immunocytochemistry for Tuj1 and DAPI. Distance from the neurosphere edge to the furthest neuron was measured in 12 directions per neurosphere and the average neuronal migration distance per neurosphere was calculated.

Next, a scratch wound assay was performed to measure neuronal recovery from injury. This assay mimics a wound environment by creating a cell-free gap or wound for cells to migrate into (Cory, 2011). ENPC were plated on a fibronectin-coated 96-well plate at a concentration of about 20,000 cells/mL at 100 μL/well in differentiation media for 7 days. Media was then changed to differentiation media without additive (*n* = 6), with 20 μM Yoda1 (*n* = 6), or with 3 μM GsMTx4 (*n* = 6), and a scratch was created. The Incucyte Live-Cell Imaging System (Sartorius, Goettingen, Germany) was used to capture images of the scratch for 10 days (240 h) post-injury. Acquired images were analyzed using the Incucyte Scratch Wound Software Analysis Module to measure and compare cell invasion into the wound. The formula for calculating the relative wound density (RWD) is given below:


$$\begin{array}{l} \%RWD\left(t\right)=100\;.\;\frac{\left(w\left(t\right)-w\left(0\right)\right)}{\left(c\left(t\right)-w\left(0\right)\right)}\\ w\left(t\right)=Density\;of\;wound\;region\;at\;time,\;\left(t\right)\\ c\left(t\right)=Density\;of\;cell\;region\;at\;time,\;\left(t\right) \end{array}$$

### Statistical analysis

All data are reported as mean ± standard deviation. Neuronal migration, neurite length, and scratch wound recovery were compared using nonparametric Mann *U-*Whitney analyses. We considered two-sides *p*-values < 0.05 to be statistically significant, and *p*-values were adjusted for multiplicity. Statistical analysis was conducted using GraphPad Prism (Version 9) and R (version 4.4.1).

## Results

### Piezo1 is expressed by enteric neurons *in vitro* and *in vivo*

**Figure 1:**
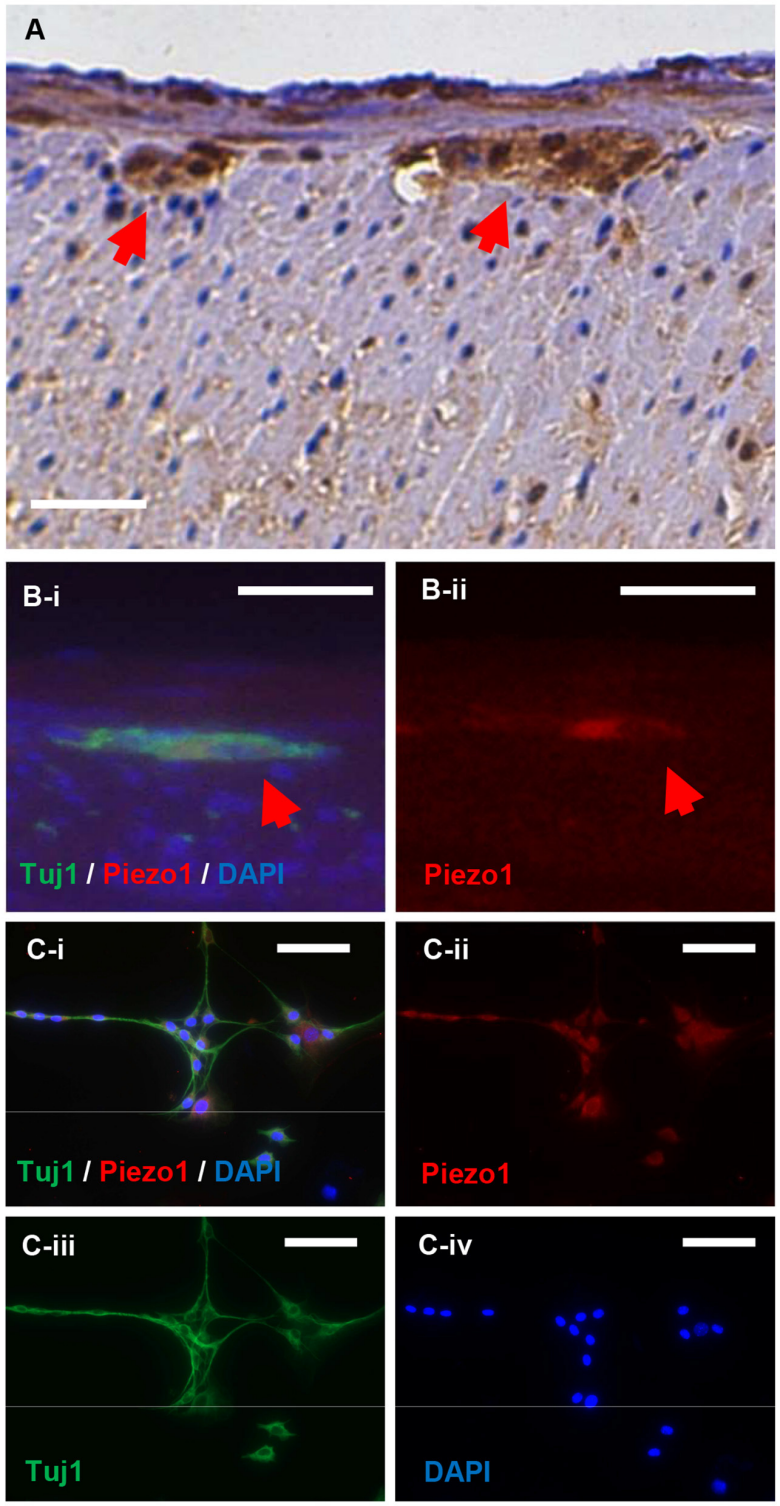
Piezo1 is expressed in enteric ganglia and by enteric neurons *in vitro*. Piezo1 in the murine colon is localized primarily to the enteric ganglia in the myenteric plexus (**A**; red arrowheads). The expression of Piezo1 co-localizes with the pan-neuronal marker, Tuj1, in normal mouse colon (**B**; red arrowheads). In cultured murine ENPC, Piezo1 expression is ubiquitously present in Tuj1+ enteric neurons **(C)**. Scale bar is 50 μm in all images.

Immunohistochemistry was performed to localize Piezo1 expression in the gut. Piezo1 appears to localize to enteric ganglia in the myenteric plexus (Figure 1A; red arrows denote myenteric plexus). Piezo1 expression appears to co-localize with the expression of the pan-neuronal marker, Tuj1, both *in vivo* in the myenteric ganglia of mouse colon (Figure 1B; red arrow denotes co-localization of Piezo1 and Tuj1 in myenteric plexus) and in cultured neurons derived from ENPC (Figure 1C). Positive immunohistochemistry for Piezo1 in the skin was confirmed as a positive control for the antibody (Supplementary Figure 1A), and negative immunofluorescence was confirmed with an isotype rabbit IgG under the same experimental setting for the control of Piezo1 staining *in vitro* (Supplementary Figure 1B). Notably, Piezo2 expression was not detectable in ENPC-derived neurons (Supplementary Figure 1C).

### Enteric neurons demonstrate significantly shorter neurite length in response to stretch

**Figure 2:**
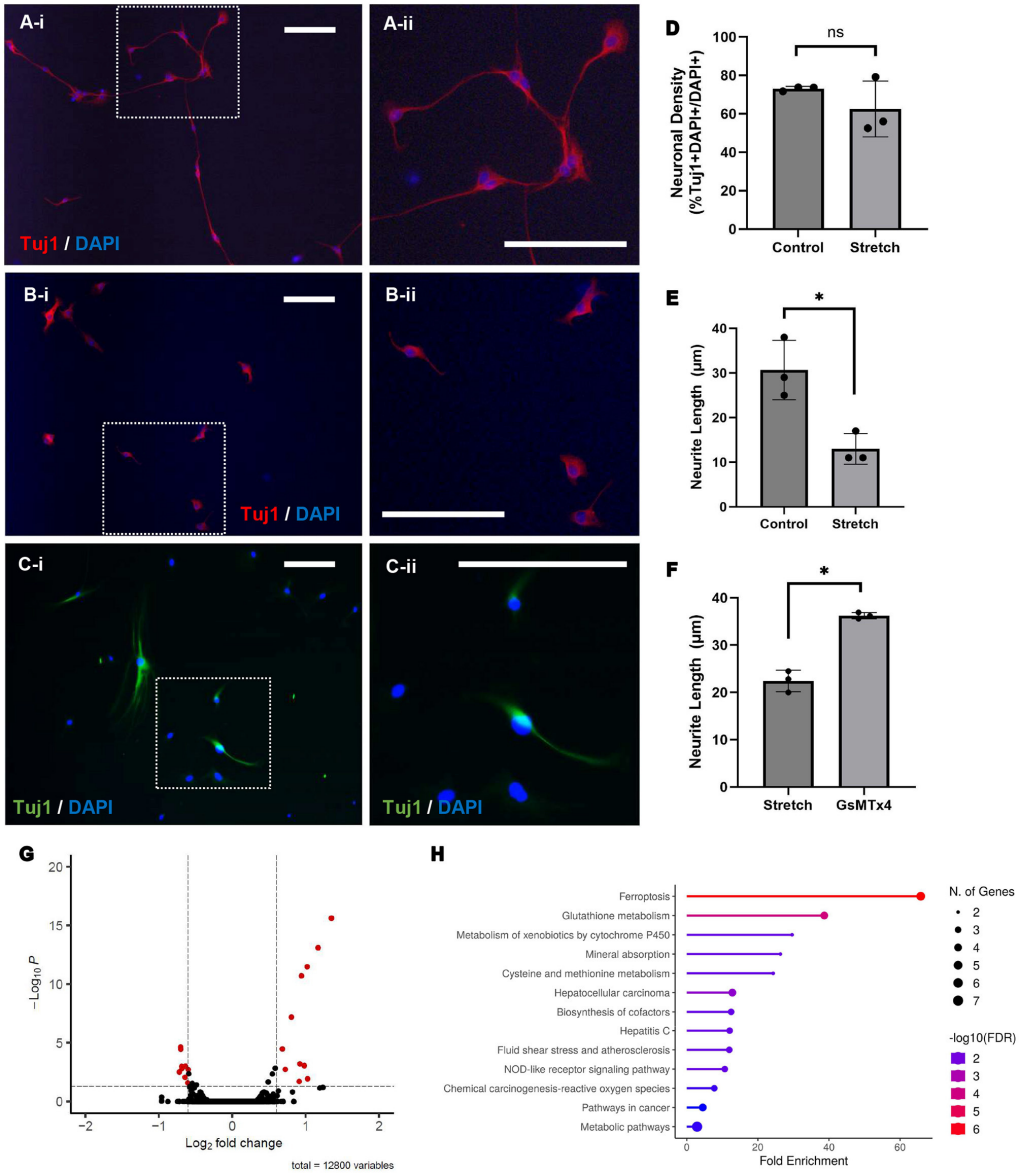
Stretch results in shorter neurite length and differential gene expression, whereas Piezo1 antagonism prevents neurite stunting in response to stretch. ENPC-derived neurons from adult mice were grown on a flexible-bottom plate and subject to up to 5% cyclic stretch. A notable difference in neuronal morphology is seen between unstretched (**A**; A-ii is magnified view of inset in A-i) and stretched (**B**; B-ii is magnified view of inset in B-i) neurons, as shown with the pan-neuronal marker, Tuj1. There was no significant difference in neuronal density between groups (**D**; ns, not significant), but neurite length was significantly shorter in stretched neurons compared to unstretched control (**E**; *=*p* < 0.05). Pre-treatment of stretched neurons with Piezo1 antagonist, GsMTx4, for 12 h prior to stretch yielded significantly longer neurites (**C**; C-ii is magnified view of inset in C-i) compared to a control group of neurons pre-treated with vehicle only prior to stretch (**F**; *= *p* < 0.05). Scale bar is 50 μm in all images. Bulk RNA sequencing was performed to compare gene expression in stretched and unstretched ENPC-derived cells. Twenty-seven genes were significantly differentially expressed in stretched cells compared to unstretched control, as demonstrated in the volcano plot **(G)**, corresponding with significant fold enrichment in 13 different KEGG pathways, as demonstrated by the lollipop chart **(H)**.

ENPC-derived neurons subjected to stretch *in vitro* were phenotypically compared to a control group of neurons at rest (Figures 2A, B; magnification shown in inset). Enteric neurons subjected to stretch appear to have stunted growth and significantly shorter average neurite length when compared to control (13 ± 3 μm vs. 31 ± 6 μm, *p* < 0.05; Figure 2E). No significant difference was observed in the percentage of neurons (% Tuj1 + DAPI + /DAPI + cells) between the control and stretched cells (73 ± 1% vs. 62 ± 15%; Figure 2D) nor in the percentage of proliferating cells (%EdU + DAPI + /DAPI + cells) during the duration of stretch (2 ± 2% vs. 1 ± 1%).

### Enteric neurons exhibit differential gene expression in response to stretch

Gene expression in ENPC-derived cells subject to stretch *in vitro* were compared ENPC-derived cells at rest using bulk RNA sequencing. Compared to the unstretched control, 12 genes were significantly downregulated and 15 genes were significantly upregulated in the stretched cells (Figure 2G). Enrichment analysis using the Kyoto Encyclopedia of Genes and Genomes (KEGG) pathway databases indicated that differentially expressed genes were involved in pathways for ferroptosis, fluid shear stress, and metabolic pathways, amongst others (Figure 2H). Notably, expression of Piezo1 and Piezo2 did not differ significantly in stretched and unstretched cells, nor did the expression of any other known mechanosensitive ion channels including: Transient Receptor Potential (TRP) channels, two-pore domain potassium (K2P) channels (e.g., TREK-1, TRAAK), voltage-gated channels (e.g., Cav1.2, Nav1.5), calcium-activated potassium channels, or focal adhesion kinase (FAK).

### Piezo1 agonism results in significantly shorter neurite length, while Piezo1 antagonism preserves neurite length in response to stretch

**Figure 3:**
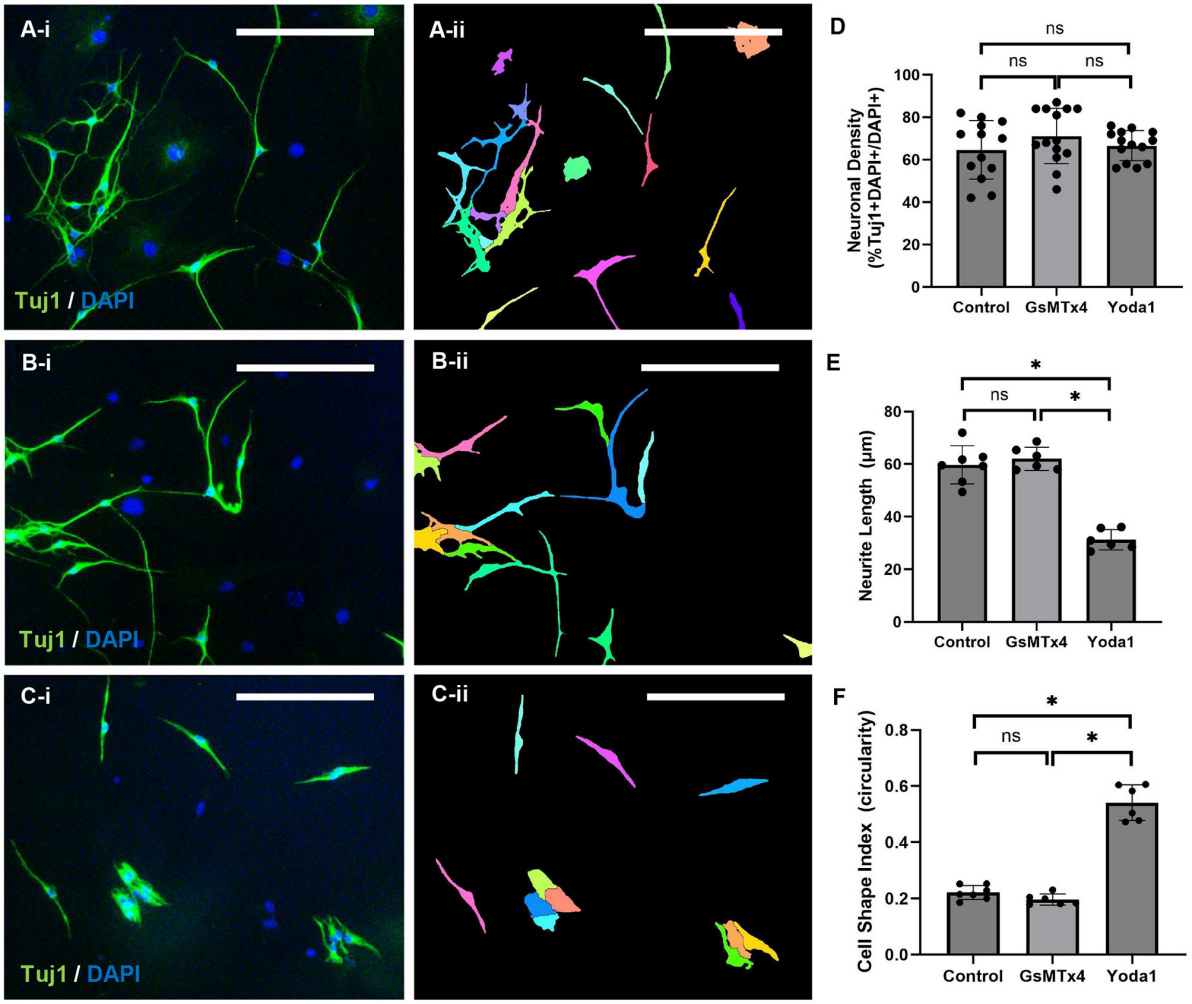
Piezo1 agonism results in significantly shorter neurite length *in vitro*. ENPC from adult mice were differentiated *in vitro* with vehicle alone **(A)**, with Piezo1 antagonist, GsMTx4 **(B)**, or with Piezo1 agonist, Yoda1 **(C)** in standard culture media under static conditions. The results are demonstrated qualitatively using the pan-neuronal marker, Tuj1 (**A–C**; A-ii through C-ii illustrates the automated cell segmentation performed in ImageJ to quantify cell shape and neurite length). Scale bar is 50 μm in all images. There were no significant differences in neuronal density between control, GsMTx4-treated, or Yoda1-treated ENPC (**D**; ns, not significant). In ENPC-derived neurons treated with Yoda1, neurite length was significantly shorter (**E**; *=*p* < 0.05) and cell circularity was significantly higher (**F**; *=*p* < 0.05) compared to both control and GsMTx4-treated cells.

The effect of Piezo1 activation and inhibition on ENPC-derived cells was measured *in vitro*. No significant difference in the percentage of neurons (% Tuj1 + DAPI + /DAPI + cells) was observed between control, 3 μM GsMTx4, and 20 μM Yoda1 (65 ± 14% vs. 71 ± 13% vs. 66 ± 7%, respectively; Figures 3A, D). Similarly, no significant difference in the percentage of neurons was observed between control, GsMTx4, and Yoda1 at reduced concentrations (Supplementary Figure 2A). A significant difference in neurite length was observed between control and 20 μM Yoda1 (60 ± 7 μm vs. 31 ± 4 μm, respectively, *p* < 0.05; Figure 3E) and between 20 μM Yoda1 and 3 μM GsMTx4 (62 ± 4 μm, *p* < 0.05; Figures 3B, E). There was a non-significant dose-dependent decrease in neurite length with increased dosage of Yoda1 (43 ± 4 μm with 10 μM Yoda1 vs. 38 ± 5 μm with 20 μM Yoda1; Supplementary Figure 2B). There was no significant difference in neurite length between control and GsMTx4 at either concentration. Cell circularity, which reflects cell morphology with 1.0 being a perfect circle and decreasing values indicating cell elongation, was also significantly different between 20 μM Yoda1 and control (0.54 ± 0.06 vs. 0.22 ± 0.03; *p* < 0.05) as well as between 20 μM Yoda1 and 3 μM GsMTx4 (0.20 ± 0.02; *p* < 0.05) but was not significantly different between control and 3 μM GsMTx4 (Figures 3C, F). Again, there was a non-significant dose-dependent increase in circularity with increased dosage of Yoda1 (0.64 ± 0.02 with 10 μM Yoda1 vs. 0.69 ± 0.02 with 20 μM Yoda1; Supplementary Figure 2C), while no difference from control was noted with either concentration of GsMTx4.

In combination, 20 μM Yoda1 and 3 μM GsMTx4 resulted in significantly reduced neuronal circularity compared to Yoda1 alone (0.62 ± 0.002 vs. 0.69 ± 0.02; *p* < 0.05) and significantly increased circularity compared to control (0.47 ± 0.03; *p* < 0.05) or GsMTx4 alone (0.43 ± 0.01; *p* < 0.05). The combination of 20 μM Yoda1 and 3 μM GsMTx4 produced a short neurite phenotype similar to Yoda1 alone (45 ± 2 μm vs. 38 ± 5 μm; *p* = NS) and significantly shorter than either control (100 ± 7 μm; *p* < 0.05) or GsMTx4 alone (104 ± 10 μm; *p* < 0.05).

To investigate if Piezo1 antagonism prevents neurite stunting in response to stretch, we measured neurite length in ENPC-derived neurons pre-treated with GsMTx4 for 12 h prior to *in vitro* stretch. Compared to control neurons treated with vehicle-only prior to stretch, there was no significant difference in the percentage of neurons (% Tuj1 + DAPI + /DAPI + cells; 89 ± 9% vs. 95 ± 5%). Intriguingly, pre-treatment with Piezo1 antagonist GsMTx4 in stretched neurons did result in a significantly longer neurite length than the stretched neurons pre-treated with vehicle only (23 ± 3 μm vs. 36 ± 1 μm, *p* < 0.05; Figures 2C, F).

### Piezo1 antagonism promotes neuronal migration, while Piezo1 agonism inhibits neuronal migration and enteric neuron recovery after injury

**Figure 4:**
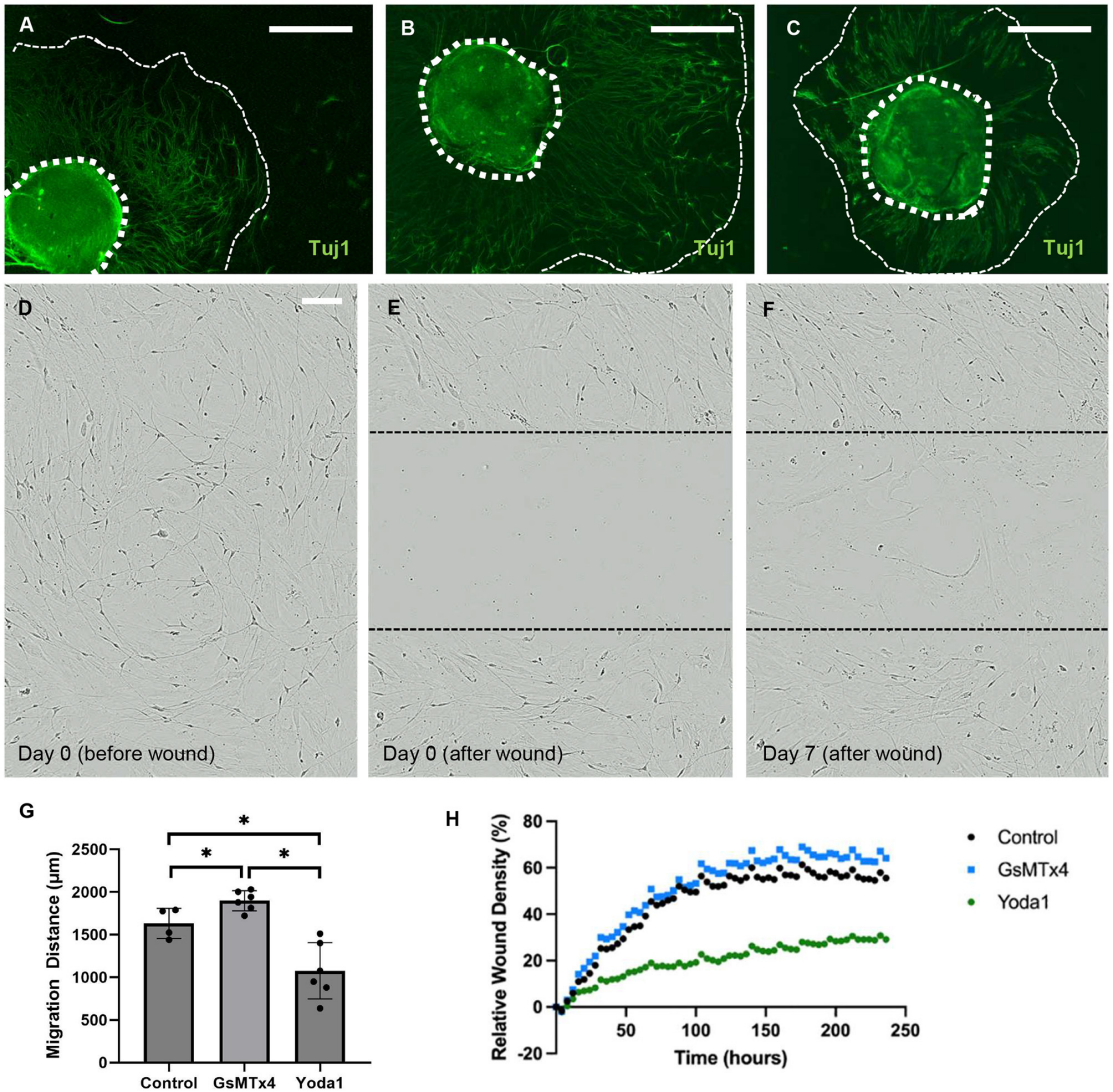
Piezo1 agonism results in significantly reduced neuronal migration *in vitro*. Enteric neurospheres were treated with vehicle alone, Piezo1 agonist (Yoda1), or Piezo1 antagonist (GsMTx4). The wavefront of neuronal migration was measured from the neurosphere edge. The wavefront of migration can be seen qualitatively using immunohistochemistry for the pan-neuronal marker, Tuj1, in representative images of each condition (**A–C**; thick dotted line marks the edge of the neurosphere, thin dashed line marks the wavefront of neuronal migration; scale bar is 500 μm). The distance of neuronal migration was significantly greater in the presence of GsMTx4 and significantly less in the presence of Yoda1 when compared to control (**G**; *=*p* < 0.05). A scratch wound assay was also used to assess ENPC migration into a wound and subsequent neuronal recovery from injury. In this assay, ENPC-derived neurons are cultured to confluence **(D)** and a linear scratch wound is created in the center of the well **(E)**. Cell in-growth into the wound is then measured over time (**F**; scale bar is 100 μm for **C–E** which shows representative images in control conditions). Relative wound density was significantly less at 10 days after injury in Yoda1-treated ENPC compared to both control (*p* < 0.05) and GsMTx4-treated (*p* < 0.05) groups **(H)**.

To investigate the impact of Piezo1 activation and inhibition on neuronal migration, we studied migration in the presence of Yoda1 and GsMTx4. Treatment with Yoda1 (Figure 4C) resulted in a significant reduction in the average migration distance of neurons compared to vehicle-only control (1,075 ± 329 μm vs. 1,632 ± 177 μm, *p* < 0.05; Figure 4A) and GsTMx4 (1,898 ± 118 μm, *p* < 0.05; Figure 4B). Conversely, treatment with GsMTx4 resulted in a significant increase in the average neuronal migration distance when compared to control (*p* < 0.05, Figure 4G).

To further investigate the impact of Piezo1 agonism and antagonism on enteric neuronal migration with respect to recovery from injury, we utilized a scratch wound assay. In this assay, ENPC were plated to confluence and allowed to differentiate for 7 days (Figure 4D) prior to creation of a scratch wound (Figure 4E) in the center of the well. Relative wound density (RWD) was then quantified over time as a measure of enteric neuronal recovery from injury (Figure 4F). We quantified the ingrowth of enteric neurons into a scratch wound in the absence of additive compared to treatment with Yoda1 or GsTMx4. There was a significantly lower relative wound density in the Yoda1 group compared to the control after 44 h (*p* < 0.05, Figure 4H) and between the Yoda1 and GsMTx4 groups after 32 h (*p* < 0.05, Figure 4H). There was no significant difference between control and GsMTx4 up to 240 h.

## Discussion

Nearly all cell types throughout the GI tract are mechanosensitive in response to a myriad of biomechanical signals. Given the critical role Piezo1 plays in mechanotransduction in the rest of the body (Bae et al., 2013; Cahalan et al., 2015), it has been hypothesized to also play an important role in the GI tract (Alcaino et al., 2017). Though previous studies did not demonstrate a change in enteric neuron mechanotransduction with activation or inhibition of Piezo1 (Mazzuoli-Weber et al., 2019), we show that Piezo1 does have a notable effect on enteric neuronal morphology and migration *in vitro* and may play a role in ENS disease pathogenesis.

Piezo1 expression in the colon was localized primarily to the myenteric ganglia, consistent with the prior literature, which described that Piezo1 is expressed in 20–70% of intrinsic enteric neurons while Piezo2 expression is limited to very few cell bodies and some neuronal processes (Mazzuoli-Weber et al., 2019; McHugh et al., 2010). Interestingly, we found that enteric neurons derived from postnatal ENPC ubiquitously expressed Piezo1 in culture. While a clinical gastrointestinal phenotype is not commonly described in human patients with Piezo1 loss of function (Lukacs et al., 2015), animal studies suggest that Piezo1 in the stomach may help regulate satiety and control food intake (Alper, 2017). We did not quantify Piezo1 expression or activation in the gut, but future studies may explore if the level of Piezo1 expression or activation may vary in different conditions or disease states, for example, in patients with obesity or binge-eating disorder.

Piezo1 may be a key player in the enteric neuronal response to stretch. Several studies have reported mechanosensitive responses in intrinsic enteric neurons to stretch, compression, shear stress, and cell edema (Dong et al., 2015; Kunze et al., 1999; Hibberd et al., 2012; Kugler et al., 2015; Kunze et al., 2000; Mazzuoli and Schemann, 2009; Spencer and Smith, 2004; Mayer and Wood, 1975). Despite the known downstream effects of biomechanical stimuli, the involved pathways are still largely unknown (Alcaino et al., 2017). Stretch and Piezo1 activation produced a similar neuronal phenotype, while the effect of stretch was abrogated by Piezo1 inhibition, suggesting a role for Piezo1 in enteric neuronal mechanotransduction. Despite this, Piezo1 expression surprisingly did not differ in stretched and unstretched neurons nor did the expression of any other known mechanoreceptors. It is very possible that receptor activation is not reflected by changes in gene expression. Examination of pathways downstream of Piezo1 and other mechanoreceptors may be more indicative of receptor activity. Importantly, KEGG pathway analysis revealed that ferroptosis pathways were significantly affected by stretch and Piezo1 is known to play an important role in iron metabolism (Ma et al., 2021). As several different mechanosensory cells and mechanoreceptors are present in the GI tract, it is unlikely that Piezo1 is the sole mediator of the enteric neuronal response to extrinsic force and future studies may explore the relationship of these various cells and circuits in response to biomechanical force. Finally, bulk RNA sequencing may lack the precision to detect differences in a heterogeneous population of ENPC-derived cells. Future studies utilizing single cell transcriptomic profiling may help elucidate mechanosensitive pathways in enteric neurons.

Piezo1 inhibition resulted in increased neuronal migration whereas Piezo1 activation resulted in decreased neuronal migration, suggesting that Piezo1 plays an important role in development of the ENS. Notably, all known Piezo1 antagonists, including the one used in this study (GsMTx4), also activate other mechanosensitive ion channels and the possibility of other off-target effects is a major limitation of our study. Transgenic mouse models may allow us to better understand the role of Piezo1 in ENS development in the future. Piezo1 is linked to cell migration in several other models with a variable effect dependent on cell type (Canales Coutino and Mayor, 2021). Piezo1 inhibition leads to increased cell migration in non-small cell lung carcinoma (Huang et al., 2019) and breast cancer (Yu et al., 2020) but decreased cell migration in gastric cancer (Zhang et al., 2018) and glioma (Chen et al., 2018). Few have studied the role of Piezo1 on migration in the ENS. Interestingly, Piezo1 is required for Xenopus cephalic neural crest migration, and loss or inactivation of the channel results in increased speed of migration (Canales Coutino and Mayor, 2021). Since the ENS is also derived from the neural crest, it is sensible that our results echo the findings from *Canales et al*. We hypothesize that Piezo1 activation may act as a brake to halt the migration of neural crest cells during development and, conversely, Piezo1 inhibition may maintain ENPC in a pro-migratory state. Future investigation of the role of Piezo1 in congenital diseases of ENS development, such as Hirschsprung disease, may reveal new pathways of disease pathogenesis and open potential avenues for therapy.

Enteric neuronal recovery from injury was also significantly impaired by Piezo1 agonism, suggesting that Piezo1 and biomechanical forces continue to impact the mature postnatal ENS. ENS injury occurs in acquired neurogastroenteropathies, such as esophageal achalasia and gastroparesis, and it is hypothesized that stretch injury may contribute to the pathogenesis of functional constipation. Taken together, our findings suggest that excess stretch or force may activate mechanosensitive pathways, such as Piezo1, to stunt ENS migration, damage enteric neurites, and contribute to GI dysfunction. Further investigation of the role of Piezo1 in enteric neuropathies is warranted and may reveal new therapies for these common GI diseases.

## Conclusion

In summary, this work describes phenotypic changes that occur in the ENS in response to biomechanical force. These changes are recapitulated by Piezo1 agonism and abrogated by Piezo1 antagonism, suggesting that this mechanoreceptor may play a role in how enteric neurons process extrinsic force. Furthermore, Piezo1 agonism results in decreased neuronal migration and recovery from injury, whereas Piezo1 antagonism results in increased neuronal migration, suggesting that Piezo1 could also play a role during ENS development and disease pathogenesis. Further investigations into the underlying molecular mechanisms and signaling pathways associated with Piezo1-mediated effects in the ENS may reveal new therapies for congenital and acquired diseases of the ENS, such as Hirschsprung disease.

## Data Availability

In accordance with open data policies, the data presented in this study are publicly available and accessible through LabArchives at doi: 10.25833/nyyp-dw94. Please contact the corresponding author for any difficulties with data accessibility.

## Supplementary material

Supplementary Figure 1 Piezo1 positive and negative controls. Piezo1 positive control exists in human melanocytes in a representative skin biopsy **(A)**. Negative control for Piezo1 with isotype control (rabbit IgG) demonstrates the absence of non-specific background immunofluorescence in Tuj1+ enteric neurons **(B)**. There was no notable immunofluorescence for Piezo2 in ENPC-derived enteric neurons **(C)**. Scale bar is 50 μm in all images.

Supplementary Figure 2 Piezo1 agonist/antagonist at varying concentrations and in combination. There was no significant difference in neuronal density between control and any tested concentrations of Yoda1 or GsMTx4 **(A)**. Neurite length was significantly shorter with both tested concentrations of Yoda1 when compared to control (**B**; ^*^=*p* < 0.05) or GsMTx4 (**B**; ^*^=*p* < 0.05). There was no difference in neurite length between control or either concentration of GsMTx4. In combination, Yoda1 and GsMTx4 resulted in significantly shorter neurite length compared to control (**B**; ^*^=*p* < 0.05). Neuronal circularity was also significantly greater with both concentrations of Yoda1 compared to control (**C**; ^*^=*p* < 0.05) while there was no difference in circularity between control or either concentration of GsMTx4. With the combination of Yoda1 and GsMTx4, neuronal circularity was significantly greater when compared to control (**C**; ^*^=*p* < 0.05) or GsMTx4 alone (**C**; ^*^=*p* < 0.05).and significantly less when compared to Yoda1 alone (**C**; ^*^=*p* < 0.05).
