# Supplementary figures and images for: Activation of mechanoreceptor Piezo1 inhibits enteric neuronal growth and migration in vitro
Source: Front Mol Neurosci. 2024 Dec 20;17:1474025. doi: 10.3389/fnmol.2024.1474025 (PMC11695422; doi:10.3389/fnmol.2024.1474025)

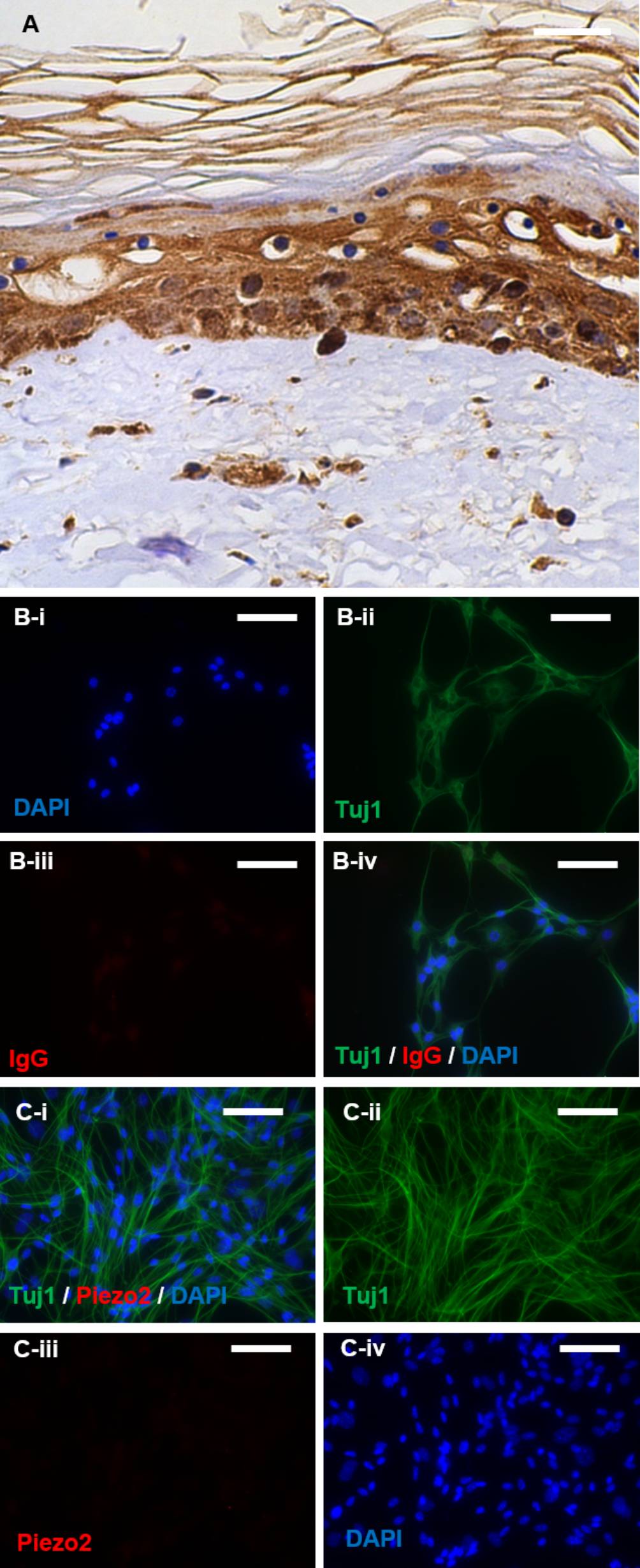

Supplement: Supplementary Figure 1 — Piezo1 positive and negative controls. Piezo1 positive control exists in human melanocytes in a representative skin biopsy (A). Negative control for Piezo1 with isotype control (rabbit IgG) demonstrates the absence of non-specific background immunofluorescence in Tuj1+ enteric neurons (B). There was no notable immunofluorescence for Piezo2 in ENPC-derived enteric neurons (C). Scale bar is 50 μm in all images. [file Image_1.jpeg]

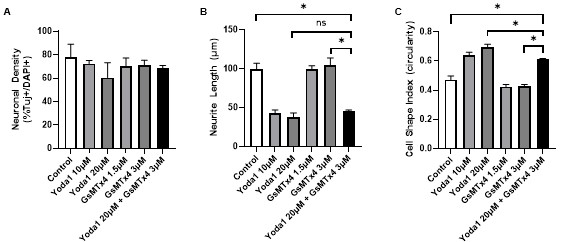

Supplement: Supplementary Figure 2 — Piezo1 agonist/antagonist at varying concentrations and in combination. There was no significant difference in neuronal density between control and any tested concentrations of Yoda1 or GsMTx4 (A). Neurite length was significantly shorter with both tested concentrations of Yoda1 when compared to control (B; *=p < 0.05) or GsMTx4 (B; *=p < 0.05). There was no difference in neurite length between control or either concentration of GsMTx4. In combination, Yoda1 and GsMTx4 resulted in significantly shorter neurite length compared to control (B; *=p < 0.05). Neuronal circularity was also significantly greater with both concentrations of Yoda1 compared to control (C; *=p < 0.05) while there was no difference in circularity between control or either concentration of GsMTx4. With the combination of Yoda1 and GsMTx4, neuronal circularity was significantly greater when compared to control (C; *=p < 0.05) or GsMTx4 alone (C; *=p < 0.05).and significantly less when compared to Yoda1 alone (C; *=p < 0.05). [file Image_2.jpeg]
